# The Predictive Value of Fibrinogen-to-Albumin Ratio for Predicting Intravenous Immunoglobulin Resistance in Kawasaki Disease: A Prospective Cohort Study

**DOI:** 10.31083/j.rcm2511421

**Published:** 2024-11-22

**Authors:** Yaru Cui, Linling Zhang, Xiaoliang Liu, Lei Liu, Kaiyu Zhou, Yimin Hua, Shuran Shao, Chuan Wang

**Affiliations:** ^1^Department of Pediatric Cardiology, West China Second University Hospital, Sichuan University, 610041 Chengdu, Sichuan, China; ^2^West China Medical School of Sichuan University, 610041 Chengdu, Sichuan, China; ^3^The Cardiac Development and Early Intervention Unit, West China Institute of Women and Children’s Health, West China Second University Hospital, Sichuan University, 610041 Chengdu, Sichuan, China; ^4^Key Laboratory of Birth Defects and Related Diseases of Women and Children (Sichuan University), Ministry of Education Chengdu, 610041 Chengdu, Sichuan, China; ^5^Key Laboratory of Development and Diseases of Women and Children of Sichuan Province, West China Second University Hospital, Sichuan University, 610041 Chengdu, Sichuan, China

**Keywords:** Kawasaki disease, prediction, fibrinogen, albumin, immunoglobulin resistance

## Abstract

**Background:**

Predicting resistance to intravenous immunoglobulin (IVIG) in the treatment of Kawasaki disease (KD) remains a focus of research. Fibrinogen and albumin in systemic inflammation play an important role. This study aims to investigate the predictive value of fibrinogen to albumin ratio (FAR) for initial IVIG resistance in patients with KD.

**Methods:**

The study prospectively recruited 962 patients with KD between July 2015 and June 2022. The serum characteristics of the two groups were compared by comparing fibrinogen and albumin, as well as other laboratory and clinical data between the IVIG-responsive and IVIG-resistant groups. Multivariate logistic regression was used to explore the relationship between FAR and IVIG resistance. Receiver operating characteristic (ROC) curves were used to determine the effectiveness of FAR in predicting initial IVIG resistance.

**Results:**

Our results demonstrated that IVIG-resistant patients had significantly higher fibrinogen levels (603.35 ± 99.00 mg/L), FAR (17.30 ± 3.31), and lower albumin (35.47 ± 5.24 g/L) compared to IVIG-responsive patients (fibrinogen 572.35 ± 145.75 mg/L; FAR 15.08 ± 4.32; albumin 38.52 ± 4.55 g/L). 15.20 was the best cut-off value of FAR for predicting initial IVIG resistance. The sensitivity was 72.5%, the specificity was 51.3%, the positive predictive value was 91.8%, and the negative predictive value was 20.0%. Multivariate logistic regression analysis, found that FAR was an independent predictor of initial IVIG resistance in KD children.

**Conclusions:**

The FAR was an independent risk factor for initial IVIG resistance, its predictive power for initial IVIG resistance exceeded that of albumin and fibrinogen alone. FAR may not be suitable as a single marker but might serve as a complementary laboratory marker to accurately predict initial IVIG resistance in KD.

## 1. Introduction

Kawasaki disease (KD) is an acute vasculitis that is the leading cause of acquired heart disease in children in the developed world. The prevalence of KD among children in China aged 0–4 years is 71.9–110.0 per 100,000 children [1]. Although high-dose intravenous immunoglobulin (IVIG) therapy has been shown to be effective in the acute phase of KD, 15% to 20% of patients develop resistance to initial IVIG therapy and develop a risk of coronary artery lesions (CALs) [2]. Therefore, it is clinically important to identify IVIG resistance in patients with KD before initiating IVIG therapy, since they may benefit from early intensive treatment such as corticosteroids [3], monoclonal antibodies [4], cytotoxic agents [5], or plasma exchange [6].

Although the etiology of KD remains unclear, systemic inflammatory responses play a crucial role in the pathogenesis and progression of KD [7]. Several studies have explored the predictive value of systemic inflammatory markers for the prognosis of IVIG-resistant patients, such as C-reactive protein [8], albumin [9], neutrophils [9], platelets [10] as well as the combination of several single markers [11]. However, none were independently found to be a valuable predictor without high sensitivities or specificities. Therefore, it is necessary to find more valuable biomarkers for predicting IVIG resistance.

Fibrinogen produced by the liver plays an important role in the inflammatory response and is an indicator of a procoagulant state. Albumin is an essential protein, rich in content, the malnutrition and inflammation inhibit synthesis. Serum albumin concentrations are associated with inflammation and the hemostasis process, so the fibrinogen and albumin ratio (FAR) not only reflects the inflammation but also reflects the blood coagulation function. Recent study has shown that FAR, as a new inflammatory marker, is closely related to acute inflammation, and is also involved in chronic and low-grade inflammation [12].

KD in the acute fever period will result in changes in blood coagulation and endothelial function, especially for IVIG-resistant patients. Therefore, IVIG resistance may reflect the more serious the inflammatory conditions. Since KD vasculitis is accompanied by increased inflammatory cells and cytokines, FAR may predict IVIG resistance. However, the predictive value of FAR for IVIG resistance in KD has not been reported. Therefore, this study aimed to verify whether FAR can be used as an effective marker to predict IVIG resistance in KD patients.

## 2. Materials and Methods

### 2.1 Study Design and Subjects

The data of patients who were treated at West China Second University Hospital from July 2015 to June 2022 were prospectively analyzed. KD was confirmed by two experienced pediatricians (at least one of whom was a KD specialist), according to the criteria recommended by the American Heart Association [13]. Data collection was performed by two experienced clinicians using a pre-coded structured questionnaire and double-checked to ensure the completeness of the data. The parents of the enrolled KD children responded to the questionnaire. The questionnaire included basic personal information, symptom description, blood test results, treatment process, and follow-up records. The institute-involved subjects have human trials approval from Sichuan University Ethics Committee (NO. 201712160121). We have obtained written informed consent from the legal guardians/relatives of the minors to consent to any publicly acceptable data included in this article.

**Fig. 1.**
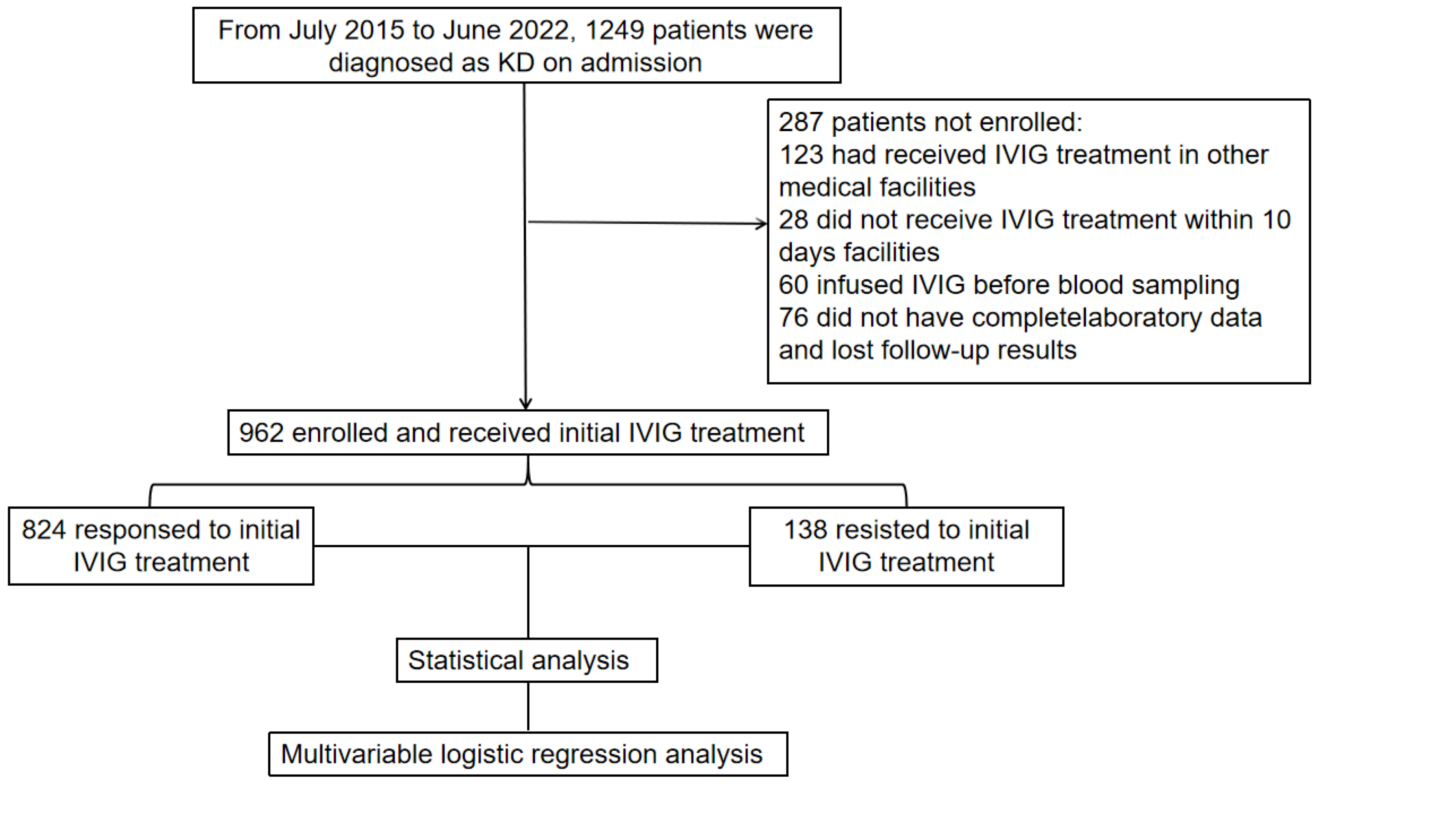
**The flowchart of our prospective study**. KD, Kawasaki disease; IVIG, intravenous immunoglobulin.

Exclusion criteria included: (1) previous oral anticoagulation or heparin therapy; (2) patients who had undergone recent surgery; (3) patients with end-stage renal disease, acute and chronic liver failure, autoimmune diseases, and malignant tumors requiring dialysis; (4) patients with known congenital or chronic blood disorders affecting the coagulation cascade. According to the above exclusion criteria, 1249 patients diagnosed with KD were first screened for participation in this study. After excluding patients who had received initial IVIG treatment at other medical institutions (n = 123), those who had not received IVIG within 10 days of fever (n = 28), and those who had started IVIG before blood collection (n = 60), a further 76 patients were excluded due to incomplete laboratory data or lack of follow-up results. Finally, a total of 962 patients were included in the study. Of the 962 patients, 824 patients (85.7%) of the initial IVIG treatment were effective, and 138 patients (14.3%) of the initial IVIG treatment were invalid. Data analysis and multivariate logistic regression analysis were subsequently performed on these two groups of patients (Fig. 1).

The collection of children began with IVIG treatment serum samples that day. The SYSMEX CA-7000 machine (SYSMEX, Tokyo, Japan) and Olympus AU5400 machine (Beckman Coulter, Tokyo, Japan) analyzer detected serum fibrinogen and serum albumin levels, collected and analyzed other laboratory indexes at the same time.

All patients received the same treatment regimen within 10 days of onset, including high-dose IVIG (2 g/kg given as a single intravenous infusion) and aspirin (30 to 50 mg/kg/day). After defervescence, aspirin dose to 3~5 mg/kg/day for 6–8 weeks. Initial IVIG resistance was defined if persistent fever (T ≥38.0 °C) or other KD clinical symptoms persisted for at least 36 hours but no more than 7 days after the first IVIG treatment and a second IVIG treatment (2 g/kg given as a single intravenous infusion) was required [14]. In addition, if there was recurrent or persistent fever after the second IVIG treatment, it was defined as repeated IVIG resistance and required the addition of intravenous prednisolone (10–30 mg/kg/day for 3 consecutive days). The oral prednisone (2 mg/kg/day) was gradually reduced after 7 days.

Coronary artery lesions (CALs) were defined based on the normalization of dimensions for body surface area as Z-scores (standard deviation units from the mean): no involvement (Z-score <2.0), dilation (Z-score ≥2.0 to <2.5), and aneurysm (Z-score ≥2.5; Z-score ≥10 for giant aneurysms) of the coronary arteries depending on the maximal internal diameters of the right, left anterior descending, and left circumflex coronary arteries. According to the study protocol, children needed regular follow-up in the cardiology clinic, first in the acute/subacute phase and then for 6 to 8 weeks. Echocardiography was performed by the same pediatric color Doppler sonographer until the lesions involving the coronary arteries had resolved. Body surface area and z scores were calculated using the Haycock [15] and Kobayashi [16] formulas, respectively.

### 2.2 Statistical Analysis

SPSS 17.0 (SPSS Inc. Chicago, IL, USA) was used for data processing and analysis. The quantitative and qualitative data were presented in different ways, as “mean ± standard deviation” and as numbers (n) versus percentages (%), respectively. The Shapiro-Wilk test and homogeneity of variance test were used after confirming that the measurement data met the criteria of normal distribution and homogeneity of variance between different groups. The differences in demographic characteristics, clinical manifestations, and laboratory data between the IVIG response group and the IVIG resistance group were compared by the chi-square test and unpaired Student’s *t*-test or Mann-Whitney U test. In addition, multivariate logistic regression analysis was used to explore the relationship between FAR and IVIG resistance. According to the receiver operating characteristic (ROC) curve, the maximum value of sensitivity and specificity Youden index was selected as the cut-off value. Setting significance as *p* < 0.05, the difference is statistically significant.

## 3. Results

### 3.1 Subjects

Of the 138 initial IVIG-resistant patients, 63 failed to respond to multiple IVIG treatments and were subsequently treated with methylprednisolone pulse therapy. No patient received treatment modalities such as infliximab, plasmapheresis, or cytotoxic agents. Among the total group, 143 patients had coronary artery lesions, 61 patients had transient pericardial effusions, 55 patients had valve regurgitation, 121 patients had cardiac enlargement, and 5 patients had ventricular systolic dysfunction. In addition, 234 patients (24.3%) were diagnosed with incomplete KD.

### 3.2 Patient Characteristics

The demographic characteristics, clinical manifestations, and laboratory data of IVIG-responsive and IVIG-resistant groups were compared between the two groups (Table 1). Compared with patients who responded to IVIG, patients who did not respond to IVIG had significantly higher levels of neutrophils, C-reactive protein, alanine aminotransferase, total bilirubin and fibrinogen, and significantly lower levels of hemoglobin, potassium, sodium and albumin (all *p* < 0.05). The resistant group had a significantly higher incidence of rash, edema & erythema of the extremities, valve regurgitation, cardiac enlargement and coronary artery lesions. The other indices showed no significant differences between two groups (all *p* > 0.05).

**Table 1.** **Comparison of the demographic characteristics, clinical and laboratory data between the IVIG-response and IVIG-resistance patients with KD in total age before initial IVIG treatment**.

		IVIG-resistance (n = 138)	IVIG-response (n = 824)	*p* value
Age (months)		38.19 ± 27.31	33.93 ± 24.76	0.066
Male (%)		83 (60.1)	471 (57.2)	0.521
Clinical manifestations				
	Rash, n (%)	114 (82.6)	588 (71.4)	0.006*
	Bilateral bulbar conjunctive injection, n (%)	129 (93.5)	749 (90.9)	0.320
	Edema & erythema of the extremities, n (%)	90 (65.2)	452 (54.9)	0.024*
	Erythema of oral and pharyngeal mucosa, n (%)	132 (95.7)	749 (90.9)	0.063
	Cervical lymphadenopathy, n (%)	74 (53.6)	376 (45.6)	0.069
	Incomplete KD, n (%)	26 (18.8)	208 (25.2)	0.105
	Pericardial effusion (%)	15 (10.9)	46 (5.6)	0.147
	Valve regurgitation (%)	42 (30.4)	13 (15.9)	0.004*
	Cardiac enlargement (%)	27 (19.6)	94 (11.4)	0.033*
	Ventricular systolic dysfunction (%)	1 (0.0)	4 (0.0)	0.401
	Coronary artery lesions (CALs), n (%)	31 (22.3)	112 (13.6)	0.014*
	Blood test from fever onset, days	4.59 ± 2.06	4.80 ± 1.91	0.235
	Fever duration before IVIG administration, days	5.54 ± 2.93	5.65 ± 1.69	0.552
Laboratory features				
	WBC count (10^9^/L)	13.73 ± 6.09	13.91 ± 4.91	0.710
	Neutrophils (%)	74.24 ± 16.03	66.27 ± 15.67	<0.001*
	Hemoglobin (g/L)	106.26 ± 13.58	109.35 ± 10.81	0.003*
	PLT count (10^9^/L)	332.70 ± 263.60	354.13 ± 168.41	0.211
	CRP (mg/L)	103.81 ± 78.23	52.99 ± 48.00	<0.001*
	ESR (mm/h)	66.87 ± 29.55	65.17 ± 28.10	0.531
	AST (IU/L)	114.63 ± 450.47	57.64 ± 111.43	0.002*
	ALT (IU/L)	116.64 ± 200.54	75.94 ± 120.74	0.001*
	ALB (g/L)	35.47 ± 5.24	38.52 ± 4.55	<0.001*
	Total bilirubin (mg/L)	18.44 ± 25.80	9.49 ± 14.44	<0.001*
	Sodium (mmol/L)	134.18 ± 3.63	136.06 ± 7.64	0.005*
	Potassium (mmol/L)	3.93 ± 0.57	4.16 ± 0.58	<0.001*
	Fibrinogen (mg/L)	603.35 ± 99.00	572.35 ± 145.75	0.002*
	FAR	17.30 ± 3.31	15.08 ± 4.32	<0.001*

The data are presented as “mean ± standard deviation” for continuous variables and as the percentage for the categorical variables. Abbreviations: IVIG, intravenous immunoglobulin; CALs, Coronary artery lesions; WBC, white blood cell; PLT, platelet; CRP, C-reactive protein; ESR, erythrocyte sedimentation rate; AST, aspartate aminotransferase; ALT, alanine aminotransferase; ALB, albumin; FAR, fibrinogen-to-albumin ratio; KD, Kawasaki disease; *, statistically significant (*p* < 0.05).

### 3.3 Analysis of Fibrinogen, Albumin, and FAR

**Fig. 2.**
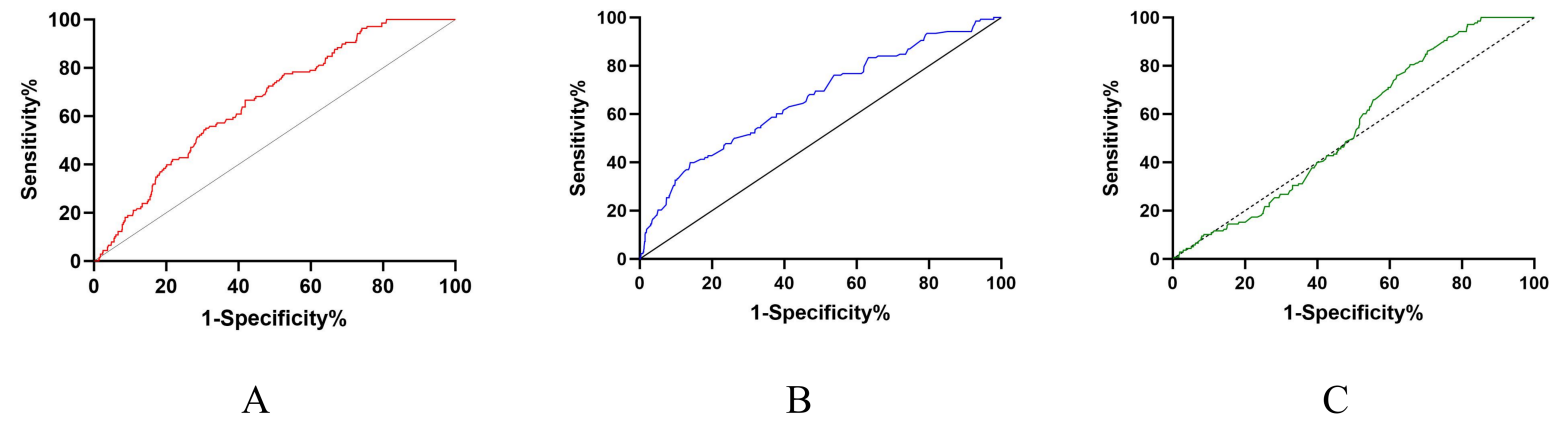
**The receiver-operating characteristic (ROC) curve for FAR (A), albumin (B) and fibrinogen (C) in predicting IVIG resistance**. FAR, fibrinogen-to-albumin ratio; IVIG, intravenous immunoglobulin.

Our results demonstrated that IVIG-resistant patients had significantly higher fibrinogen levels (603.35 ± 99.00 mg/L), FAR (17.30 ± 3.31), and lower albumin (35.47 ± 5.24g/L) compared to IVIG-responsive patients (fibrinogen 572.35 ± 145.75 mg/L; FAR 115.08 ± 4.32; albumin 38.52 ± 4.55). The best FAR cutoff value for predicting initial IVIG resistance was 15.20, yielding a sensitivity of 72.5%, a specificity of 51.3%, a positive predictive value of 91.8%, and a negative predictive value of 20.0% (Table 2). The area under the ROC curve was 0.58 (95% CI [3.10 (2.03–4.73)], *p* < 0.001) (Fig. 2A). In terms of albumin, the best cutoff for predicting IVIG resistance was 33.65 g/L, and the corresponding sensitivity, specificity, positive predictive value, and negative predictive value were 40.6%, 85.8%, 32.4%, and 89.6%, respectively. The area under the ROC curve was 0.68 (95% CI [4.13 (2.79–6.11)], *p* < 0.001) (Fig. 2B). The best fibrinogen cutoff value for predicting initial IVIG resistance was 447.5 mg/L, yielding a sensitivity of 97.8%, a specificity of 15.9%, a positive predictive value of 97.8%, and a negative predictive value of 16.3% (Table 2). The area under the ROC curve was 0.57 (95% CI [4.96 (1.96–12.61)], *p* = 0.069) (Fig. 2C).

**Table 2.** **The validity of FAR, fibrinogen and albumin cut-off values in predicting initial IVIG resistance for the total group**.

Initial IVIG resistance	Diagnostic test	Gold standard			Sen	Spe	PPV	NPV	Diagnostic accuracy	OR (95% CI)	*p*
Total group (n = 962)	FAR ≥15.20	positive	100	401	72.5	51.3	91.8	20.0	0.58	3.10 (2.03–4.73)	<0.001*
		negative	38	423							
	Albumin ≤33.65 g/L	positive	56	117	40.6	85.8	32.4	89.6	0.68	3.11 (2.31–4.19)	<0.001*
		negative	82	707							
	Fibrinogen ≥447.5 mg/L	positive	135	693	97.8	15.9	97.8	16.3	0.45	8.51 (2.70–27.11)	<0.001*
		negative	3	131							

Abbreviations: IVIG, intravenous immunoglobulin; Sen, sensitivity; Spe, specificity; PPV, positive predictive value; NPV, negative predictive value; FAR, fibrinogen-to-albumin ratio; OR, odds ratio; CI, confidence interval; *, statistically significant (*p* < 0.05).

### 3.4 Multivariable Logistic Regression Analysis

Based on the results of the multiple logistic regression analysis in Table 3, we explored whether FAR was an independent risk factor for KD IVIG resistance. Statistically significant variables identified as confounding factors included neutrophils (N)%, hemoglobin, CRP, ALT, AST, albumin, fibrinogen, K+ and Na+ levels. The results showed that FAR ≥15.20 was an independent risk factor for initial IVIG resistance (OR: 3.10 95% CI (2.03–4.73), *p* < 0.001) (Table 3).

**Table 3.** **A multivariate logistic regression model from univariate analysis for initial IVIG resistance in patients with KD**.

Variates	β	SE	Walds	*p* value	OR	95% CI
N%	–0.017	0.008	3.819	0.051	0.894	0.99–1.00
Hemoglobin	0.002	0.010	0.042	0.839	1.000	0.98–1.02
CRP	–0.004	0.002	3.258	0.071	0.996	0.99–1.00
ALT	0.000	0.001	0.004	0948	1.000	0.99–1.00
AST	–0.002	0.001	2.171	0.141	0.998	0.99–1.00
TBil	–0.014	0.005	6.477	0.011*	0.986	0.97–0.98
Albumin	0.360	0.059	37.68	<0.001*	1.433	1.27–1.61
Fibrinogen	–0.017	0.003	29.11	<0.001*	0.980	0.97–0.98
Na+	0.013	0.011	1.284	0.257	1.013	0.99–1.03
K+	0.201	0.202	1.393	0.320	1.222	0.82–1.81
FAR	0.589	0.110	28.71	<0.001*	1.802	1.45–2.23

Abbreviations: N, neutrophils; CRP, C-reactive protein; ALT, alanine aminotransferase; AST, aspartate aminotransferase; TBil, total bilirubin; Na, sodium; K, potassium; FAR, fibrinogen-to-albumin ratio; IVIG, intravenous immunoglobulin; KD, Kawasaki disease; SE, standard error; OR, odds ratio; CI, confidence interval; *, statistically significant (*p* < 0.05).

## 4. Discussion

In this study, we prospectively explored the predictive value of FAR for IVIG resistance in KD using the largest sample size to date. As FAR as we know, the queue for the first time to explore the FAR to predict the effectiveness of IVIG resistance. In addition, we not only evaluate the sensitivity and specificity, but also evaluate the positive predictive value (PPV) and positive predictive value (NPV). Our main finding was a significantly elevated FAR in KD children who did not respond to initial IVIG therapy. FAR is KD children’s initial IVIG therapy independent risk factor for the development of IVIG resistance. FAR the best critical value of 15.20 to predict IVIG resistance, sensitivity is 0.72, specificity is 0.51 (Table 2). Given the very low sensitivity of albumin and the very low specificity of fibrinogen, FAR is superior to albumin and fibrinogen in identifying initial IVIG resistance.

Almost all causes of the systemic inflammatory response are closely related to a certain degree of coagulation activation [17]. Fibrinogen is a large, complex, fibrous glycoprotein, which plays a vital role in clot formation and also acts as a proinflammatory cytokine in the inflammatory environment of peripheral blood and reflects the development of inflammation [18]. Under normal conditions, the concentration of fibrinogen in the blood should be between 200 mg/dL and 400 mg/dL [19]. However, if the body develops a disease process, such as cancer, vascular disease, injury, infection, or inflammation, the fibrinogen concentration is significantly increased [20]. It has been suggested that cancer-related inflammation could stimulate the hemostatic system to promote a prothrombotic tendency and trigger the generation of fibrinogen [21, 22]. Evidence for a marked increase in fibrinogen during the early phase of KD has come from US investigators [23]. Chen *et al*. [24] studied 20 children with KD compared with 10 healthy children, and found hyperfibrinogenemia in patients with KD. A similar result was also found in our study, in which the fibrinogen level was significantly higher in IVIG resistant patients. The higher fibrinogen levels seen in these patients may be indicative of more severe inflammation and increased coagulation activation.

It has also been proposed that vascular leakage may be a key feature of KD pathophysiology [25]. Albumin is the most abundant protein in plasma and has a variety of functions. Study has shown that a low concentration of albumin and the increased inflammatory response as well the increased release of proinflammatory cytokines, may contribute to the progression of KD [26]. It has been reported that hypoalbuminemia is the result of the combined effects of inflammation and inadequate protein in patients with critical diseases [27, 28]. For these reasons, albumin, a negative acute-phase reactant, may be considered a surrogate marker of serious infectious disease. In children with KD, serum albumin increases permeability and leakage of fluid during the acute stage. Previous research has shown that acute stage albumin levels are decreased in intensive Kawasaki disease [29]. Some systems assessing the risk of IVIG resistance in Kawasaki disease use albumin levels below 3.5 g/dL as the reference standard [9]. In this study, we also found that the albumin levels in the resistant group were significantly lower than the IVIG-responsive group. However, consistent with previous studies, this marker has a low sensitivity and suggests that combination with other specific indicators might be more valuable and accurate.

FAR is associated with microinflammation and has recently been recognized as a novel marker of inflammation. Previous study has shown that FAR has a prognostic significance in predicting the severity of ST-segment elevation myocardial infarction in patients [30]. In addition, Zou *et al*. [31] found that a high FAR was an adverse prognostic biomarker for chronic lymphocytic leukemia, highlighting the possibility that severe inflammation might accelerate and exacerbate the development of the disease. Furthermore, Tan *et al*. [32] reported that in malignant diseases, FAR has a positive correlation with common inflammatory markers such as C-reactive protein and the ratio of neutrophils and lymphocytes, which suggest that FAR might be used to evaluate the state of the inflammatory response. Therefore, FAR is not only associated with coagulation and nutrition, but also with inflammation. Therefore, it appears to be reasonable to explore the diagnostic efficacy of FAR for IVIG resistance, based on its ability to predict the degree of systemic inflammation.

According to the results of previous studies [30, 31, 32], FAR is considered to be a strong prognostic indicator in patients with inflammatory diseases. Our study found that FAR is an independent risk factor for resistance to initial IVIG treatment in patients with KD, and its predictive ability is more valuable and accurate than fibrinogen or albumin alone. In children with IVIG resistance, neutrophils, C-reactive protein (CRP), alanine aminotransferase (ALT), aspartate aminotransferase (AST), total bilirubin (TBil) level increased significantly, while hemoglobin, Na+, K+ level is significantly lower. However, the predictive abilities of the above parameters as a single marker were not as good as FAR (**Supplementary Table 1**). Furthermore, after FAR is integrated with the above clinical and laboratory markers, respectively. The combination did not enhance the predictive values with extremely low sensitivities (**Supplementary Table 2**). So, although FAR in the prediction of IVIG resistant performance is good, its specificity is relatively low, meaning it cannot identify all IVIG reactions in patients. Simple FAR ratios derived from routine blood tests may be a cost-effective alternative and provide additional information for predicting IVIG resistance in patients with KD. However, FAR does not accurately identify all patients with initial IVIG resistance, and there may be some false negative and false positive results. Therefore, we believe that predictive models incorporating other specific measures rather than clinical and routine laboratory variables may perform better. In order to better understand the mechanism of IVIG-resistant KD, there is a need for a larger sample size of prospective studies.

There are some limitations to this study. First of all, this study involved only a single institution, because our hospital is the largest pediatric medical center in southwest China, so there may be some selection bias because the children we admitted may be more severe. Second, the results may apply only to children with KD who received standard IVIG (2 g/kg) for 10 days before the onset of fever, since we restricted the days to get the initial IVIG treatment to within 10 days. Third, since only fibrinogen serum concentration levels before IVIG administration were examined, the prognostic role of FAR after IVIG administration could not be analyzed. Thus, the predictive validity for repeated IVIG resistance remains unknown. These limitations might influence the generalizability of our results. The present study explored the predictive value of FAR for initial resistance to IVIG on the basis of a large-scale, prospective study. The level of FAR is significantly increased in IVIG-resistant KD children, which can be used as a laboratory marker to predict IVIG resistance. Given the unknown origin of KD, these findings may be helpful when developing therapeutic strategies for the treatment of KD in children.

## 5. Conclusions

The FAR was an independent risk factor for initial IVIG resistance, its predictive power for initial IVIG resistance exceeded that of albumin and fibrinogen alone. FAR may not be suitable as a single marker but might serve as a complementary laboratory marker to accurately predict initial IVIG resistance in KD.

## Availability of Data and Materials

All data generated or analyzed during this study are included in this published article and the supplementary files.

## Supplementary Material

Supplementary material associated with this article can be found, in the online version, at https://doi.org/10.31083/j.rcm2511421.
