# Supplementary material for: The Predictive Value of Fibrinogen-to-Albumin Ratio for Predicting Intravenous Immunoglobulin Resistance in Kawasaki Disease: A Prospective Cohort Study
Source: Rev Cardiovasc Med. 2024 Nov 22;25(11):421. doi: 10.31083/j.rcm2511421 (PMC11607511; doi:10.31083/j.rcm2511421)
Supplement: Supplementary file 1 [file 2153-8174-25-11-421-s1.zip › Supplementary Table 1.docx]

Supplementary table1. Predictive ability of univariate indicators in initial IVIG resistance prediction for patients with KD.

| Diagnostic test | Sen(%) | Spe(%) | PPV(%) | NPV(%) | Diagnostic accuracy | OR (95%CI) | P |
| --- | --- | --- | --- | --- | --- | --- | --- |
| Neutrophils≥81.35% | 90.49 | 32.18 | 83.33 | 47.45 | 0.78 | 4.51(3.08-6.62) | <0.001* |
| Hemoglobin≤102.5g/L | 87.45 | 18.75 | 74.67 | 35.29 | 0.69 | 1.61(1.09-2.36) | 0.015* |
| CRP≥61.9mg/L | 92.67 | 18.82 | 43.17 | 79.41 | 0.48 | 2.93(1.89-4.54) | 0.005* |
| AST≥40.5U/L | 88.89 | 20.48 | 67.96 | 49.28 | 0.65 | 2.06(1.43-2.97) | <0.001* |
| ALT≥52.5U/L | 89.26 | 20.22 | 64.56 | 53.62 | 0.63 | 2.11(1.46-3.03) | <0.001* |
| ALB≤33.65g/L | 89.61 | 32.37 | 85.80 | 40.58 | 0.79 | 4.13(2.79-6.11) | <0.001* |
| Total bilirubin≥8.75mg/dl | 75.63 | 10.25 | 25.61 | 50.72 | 0.29 | 0.35(0.25-0.51) | <0.001* |
| Sodium≤136.05mmol/L | 92.90 | 22.62 | 57.93 | 73.53 | 0.60 | 3.83(2.55-5.74) | <0.001* |
| Potassium≤3.70mmol/L | 89.10 | 25.93 | 80.34 | 41.18 | 0.75 | 2.86(1.95-4.20) | <0.001* |

Abbreviations: CRP, C-reactive protein; AST, aspartate aminotransferase; ALT, alanine aminotransferase; FAR, fibrinogen-to-albumin ratio

*Statistically significant (p<0.05)
