# Supplementary material for: The Predictive Value of Fibrinogen-to-Albumin Ratio for Predicting Intravenous Immunoglobulin Resistance in Kawasaki Disease: A Prospective Cohort Study
Source: Rev Cardiovasc Med. 2024 Nov 22;25(11):421. doi: 10.31083/j.rcm2511421 (PMC11607511; doi:10.31083/j.rcm2511421)
Supplement: Supplementary file 1 [file 2153-8174-25-11-421-s1.zip › Supplementary Table 2.docx]

Supplementary table2. Sensitivity, specificity, PPV, NPV, diagnostic accuracy of cut-off values of conventional indicators in initial IVIG resistance prediction.

| Diagnostic test | Sen(%) | Spe(%) | PPV(%) | NPV(%) | Diagnostic accuracy | OR (95%CI) | P |
| --- | --- | --- | --- | --- | --- | --- | --- |
| Neutrophils≥81.35%  FAR≥15.20 | 43.1 | 88.3 | 90.3 | 38.1 | 0.86 | 5.72(3.84-8.53) | <0.001* |
|  |  |  |  |  |  |  |  |
| Hemoglobin≤102.5g/L  FAR≥15.20 | 30.1 | 85.1 | 88.0 | 25.2 | 0.77 | 2.47(1.64-3.74) | <0.001* |
|  |  |  |  |  |  |  |  |
| CRP≥61.9mg/L  FAR≥15.20 | 58.8 | 64.5 | 90.4 | 21.6 | 0.64 | 2.60(1.79-3.76) | <0.001* |
|  |  |  |  |  |  |  |  |
| AST≥40.5U/L  FAR≥15.20 | 36.2 | 84.2 | 88.7 | 27.8 | 0.77 | 3.03(2.05-4.50) | <0.001* |
| ALT≥52.5U/L  FAR≥15.20 | 42.8 | 79.1 | 89.2 | 25.5 | 0.74 | 2.83(1.94-4.13) | <0.001* |
|  |  |  |  |  |  |  |  |
| Total bilirubin≥8.75mg/dl  FAR≥15.20 | 37.0 | 86.5 | 89.1 | 31.5 | 0.79 | 3.77(2.53-5.61) | <0.001* |
|  |  |  |  |  |  |  |  |
| Sodium≤136.05mmol/L  FAR≥15.20 | 58.1 | 75.0 | 91.5 | 28.3 | 0.73 | 4.25(2.92-6.19) | <0.001* |
|  |  |  |  |  |  |  |  |
| Potassium≤3.70mmol/L  FAR≥15.20 | 36.8 | 86.7 | 89.1 | 31.6 | 0.80 | 3.80(2.54-5.69) | <0.001* |
|  |  |  |  |  |  |  |  |
